# Evaluation of Energy Expenditure and Oxidation of Energy Substrates in Adult Males after Intake of Meals with Varying Fat and Carbohydrate Content

**DOI:** 10.3390/nu10050627

**Published:** 2018-05-16

**Authors:** Edyta Adamska-Patruno, Lucyna Ostrowska, Anna Golonko, Barbara Pietraszewska, Joanna Goscik, Adam Kretowski, Maria Gorska

**Affiliations:** 1 Clinical Research Centre, Medical University of Bialystok, Bialystok 15-276, Poland; joanna.goscik@umb.edu.pl (J.G.); adamkretowski@wp.pl (A.K.); 2 Department of Dietetics and Clinical Nutrition, Medical University of Bialystok, Bialystok 15-054, Poland; lucyna.ostrowska@umb.edu.pl (L.O.); anna.golonko@umb.edu.pl (A.G.); b.pietraszewska@o2.pl (B.P.); 3 Department of Endocrinology, Diabetology and Internal Medicine, Medical University of Bialystok, Bialystok 15-276, Poland; mgorska25@wp.pl

**Keywords:** obesity, adults, energy expenditure, glucose utilization, lipid oxidation

## Abstract

Obesity is a result of positive energy balance. The aim of this study was to measure (in crossover trials) the energy expenditure and oxidation of glucose and lipids, both at the fasting state and after an intake of meals with a varying macronutrient content, in normal-weight and overweight/obese people. In the study, 46 healthy adult males (23 with normal body weight and 23 overweight/obese), aged 21–58, were examined. During two consecutive visits, subjects received isocaloric standardized meals (450 kcal) with different content of basic nutrients. Resting metabolic rate and carbohydrate and fat utilization were evaluated during the fasting state and postprandially, using an indirect calorimetry method. Energy expenditure was higher in people with normal body weight and slightly higher after the high-carbohydrate meal. In overweight/obese people, increased expenditure was noted after normo-carbohydrate meal intake. The high-fat meal induced lower postprandial thermal response compared to a high-carbohydrate meal, both in people with normal body weight and in overweight/obese men. Glucose utilization was higher after the high-carbohydrate meal, and it was higher in the normal body weight group than in overweight/obese people. In addition, overweight/obese people showed a lower level of fatty acid oxidation under fasting conditions which, together with limited ability to oxidize energy substrates, depending on their availability, indicates that these people are characterized by lower metabolic flexibility.

## 1. Introduction

Obesity is a chronic disease resulting from dysfunction of the mechanisms that regulate energy homoeostasis. The knowledge of the causes and the basis for excessive accumulation of adipose tissue has expanded rapidly over the past several years, however, the pathophysiological mechanisms which lead to its development, due to the complex etiology of obesity, are not fully understood. Disturbances of energy balance are influenced by a number of factors, genetic and environmental, and their mutual interactions appear to play a key role [1]. Indeed, not all individuals who are exposed to obesogenic environmental factors and have genetic susceptibility become obese [2]. Obesity, in general, is associated with insulin resistance and with disturbances in glucose and lipid metabolism, such as diminished glucose utilization, while fatty acids become the predominant fuel source in muscle [3]. Since all of the mentioned metabolic disturbances may lead to type 2 diabetes development, investigations of the impact of diet are of great importance for better understanding the mechanisms that lead to metabolic disorders.

Energy balance is regulated by the central nervous system. The arcuate nucleus of the hypothalamus receives central and peripheral signals further transmitted to the paraventricular nucleus and other structures, which are involved in the regulation of energy homoeostasis—affecting nutritional behavior and energy expenditure. Peripheral signals (mechanical, metabolic, and hormonal) influence energy expenditure, energy substrates utilization, as well as lipolysis and lipogenesis processes. The synthesis and/or secretion of these factors may depend on the current energy state of the body, whereas in the postprandial period it may depend also on the nutritional composition of food [4,5], and even drinking water may have an impact on energy expenditure and substrate utilization [6]. Moreover, because many hours per day are spent in the postprandial state, the metabolic consequences of meal intake may have a substantial impact on the risk of metabolic disorders. It is also suggested that postprandial dysmetabolism may be associated with increased risk of numerous disturbances and disorders, and concentrations of some postprandial factors seem to be better predictors of some diseases (for example, cardiovascular disease risk) than the fasting blood levels of these factors [7]. A higher diet-induced thermogenesis as a response to meal intake (postprandial energy expenditure) can also imply that less energy is available for storage in adipose tissue and other structures, hence, such a meal may be less conducive to weight gain [8].

The role of individual nutrients has not been fully elucidated yet. In addition, the metabolic response to meal intake may vary in people with normal body weight compared to people who are obese. There are many discrepancies among reports, and some authors observed differences between normal-weight and obese people, but some studies did not confirm this. Moreover, results from studies on meal composition often report the opposite effects [9,10,11,12,13], which indicates the need for further investigations. Therefore, the aim of our study was to evaluate (in a crossover trial) energy expenditure and energy substrate utilization at the fasting state and after consumption of meals with varying nutrient content, in adult males with different body weight.

## 2. Methods

The study was conducted among Caucasian men of Polish origin, who were recruited to the meal test studies for our other experiments [14,15,16]. Therefore, study population, study procedures, and statistical analysis have been described in detail previously. The study population characteristics are presented in Table 1. Subjects were instructed to maintain their regular lifestyle throughout the study and to avoid coffee, alcohol, and excessive physical exercise three days before test. The study protocol was approved by local Ethics Committee (Medical University of Bialystok, Poland, R-I-002/35/2009). Participating patients were informed about the aim of the study and voluntarily agreed to participate in the study.

**Figure 1:**
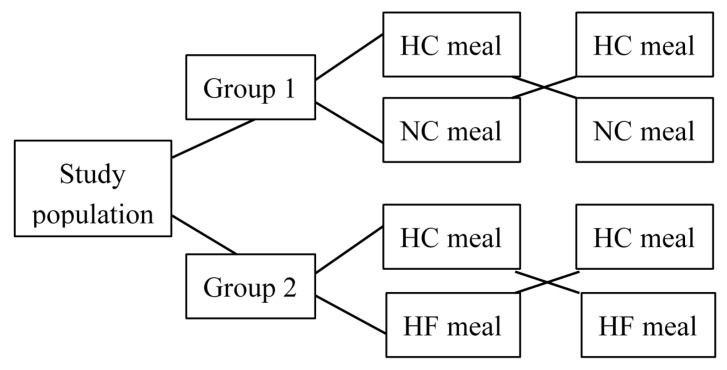
The study design. HC—high-carbohydrate meal; NC—normal-carbohydrate meal; HF—high-fat meal.

Based on BMI (Body Mass Index), the men were divided into two groups—people with normal body weight (N) and people who were overweight/obese (O/O). Subsequently, the subjects were randomly assigned (randomization using a simple draw without returning) to one of two groups: (1) Group I (G1)—11 men with normal body weight (N1) and 12 men who were overweight/obese (O/O1); (2) Group II (G2)—12 men with normal body weight (N2) and 11 men who were overweight/obese (O/O2). The crossover method was using to carry out the study (Figure 1). People from G1 received a standardized (450 kcal) high-carbohydrate meal (HC—Nutridrink Fat Free, Nutricia, Opole, Poland) and after 1–2 weeks they received an isocaloric (450 kcal) normo-carbohydrate meal (NC—Cubitan, Nutricia, Opole, Poland). Similarly, men from G2 received standardized (450 kcal) high-carbohydrate meal (HC—Nutridrink Fat Free, Nutricia, Opole, Poland) and after 1–2 weeks they received an isocaloric (450 kcal) high-fat meal (HF—Calogen, Nutricia, Opole, Poland) meal. The macronutrient meal compositions are presented in Table 2.

### 2.1. Study Procedures and Indirect Calorimetry Measurements

After an overnight fast, the participants arrived at the laboratory at 08:00 on the test day. Upon arrival at the laboratory, subjects were positioned in bed in a quiet room, with thermoneutral conditions (22–25 °C), to rest for at least 30 min, followed by performance of fasting measurement of metabolic rate and substrates utilization. Postprandial resting energy expenditure (REE) and substrates utilization were measured 60, 120, 180, and 240 min following the meal challenge. The energy expenditure and substrates utilization were evaluated by computed open-circuit indirect calorimetry—which is the noninvasive recommended method to measure REE [17,18]—based on the consumption of O2 and the production of CO2. The measures of resting oxygen uptake and resting carbon dioxide production were performed by a ventilated canopy Vmax Encore 29n System (Viasys HealthCare, Yorba Linda, CA, USA), which is the most valid instrument for both RMR (Resting Metabolic Rate) and RER (Respiratory Exchange Ratio) assessment [19]. Calibrations with gases with known and certified CO_2_ and O_2_ composition were completed before starting the assessments, and measurements were standardized by internal guidelines. The measurements were performed for 30 min each and were expressed as mg/ffm/min values. The system provided calculated results of energy expenditure and substrates utilization.

### 2.2. Statistical Analysis

Descriptive statistics, including mean and its standard error, were calculated for all numerical features representing concentrations of interest, which underwent further, consecutive steps of the analysis. The aim of the study was to check whether postprandial metabolic responses differ significantly (in terms of statistical significance) when types of meals and patients’ characteristic were used as a grouping factor. Two main null hypotheses were stated: (1) type of meal (identified by the main ingredient) has no influence on postprandial metabolic response in normal-weight and overweight/obese patients (groups of patients analyzed separately), (2) there is no statistically significant difference in postprandial metabolic response to a particular meal in normal-weight and overweight/obese patients (types of meals analyzed separately). The first hypothesis was verified for the following pairs of meals: HC vs. NC, HC vs. HF. The procedure was conducted twice—for normal-weight and overweight/obese patients—and, since both meals were given to the same patients, the lack of independence was taken into consideration, resulting in the choice of statistical tests. Either one-way ANOVA (analysis of variance) [20] or Wilcoxon signed-rank test [21] (both for paired samples) was carried out, depending on fulfillment of the condition of the normality of the variables’ distribution, which was checked with the Shapiro–Wilk test [22]. The second hypothesis was verified for investigated types of meals: HC, NC, and HF. The goal was to investigate whether there are statistically significant differences in postprandial metabolic response between normal-weight and overweight/obese subjects. One-way ANOVA or Wilcoxon rank-sum test [23] (both for unpaired samples)—depending on fulfillment of the condition of the normality of the variables’ distribution and the homogeneity of variances—were used to test the stated hypothesis. The homogeneity of variances was verified with the Levene test [24]. To address the issue of multiple hypothesis testing, the false discovery rate [25] *p*-value adjustment method was used. For all calculations, the alpha level was set at 0.05. The areas under the curve (AUCs) were calculated using the trapezoidal method and underwent the same analysis schema as the rest of the features.

## 3. Results

In Groups I and II, the energy expenditure was evaluated after standardized isocaloric meal intake: a high-carbohydrate (HC), a normo-carbohydrate (NC), and a high-fat (HF) meal. In Group I, in subjects with normal body weight (N1), no significant differences in energy expenditure after the HC and NC meal intake were observed, while in overweight/obese people (O/O1) there was a tendency towards a higher energy expenditure 60 min after NC meal consumption, compared to the HC meal (Table 3). The tendency towards a higher value of the area under the energy expenditure curve was noticed for post-intake of the HC meal compared to the NC meal in people with normal body weight, which was not observed in overweight/obese men.

In Group II, in subjects with normal body weight (N2), significantly higher energy expenditure was shown 60 min after the HC meal vs. HF meal intake (0.0172 ± 0.0008 kcal/kg/min vs. 0.0152 ± 0.0005 kcal/kg/min, *p* = 0.01) and this tendency was also observed at 120 min (Table 3). In overweight/obese men (O/O2), energy expenditure was significantly higher after the HC meal vs. HF meal at 60 min of the test (0.0145 ± 0.0005 vs. 0.0128 ± 0.0004, *p* = 0.0008) (Table 3). Both in normal-weight and overweight/obese subjects, the area under the energy expenditure curves did not significantly differ after high-carbohydrate and high-fat meal intake.

**Figure 2:**
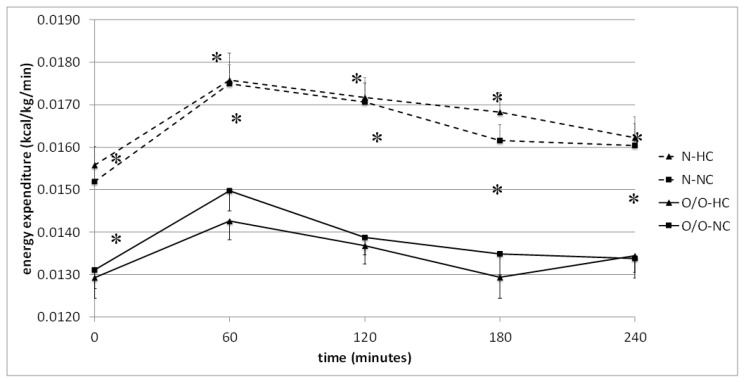
Energy expenditure (kcal/kg/min) in people with normal body weight (N, the broken line) and overweight/obese people (O/O, the solid line) in fasting state (time 0 min) and after consumption (time 60–240 min) of a high-carbohydrate meal (HC) and a normo-carbohydrate meal (NC). The results are presented as mean values ± SE. The comparison between study groups N and O/O * *p* < 0.05; the comparison between meals HC and NC.

**Figure 3:**
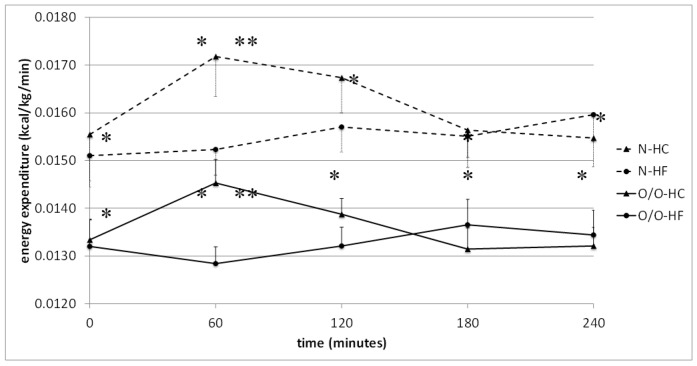
Energy expenditure (kcal/kg/min) in people with normal body weight (N, the broken line) and overweight/obese people (O/O, the solid line) in fasting state (time 0 min) and after consumption (time 60–240 min) of a high-carbohydrate meal (HC) and a high-fat meal (HF). The results are presented as mean values ± SE. The comparison between study groups N and O/O * *p* < 0.05; the comparison between meals HC and HF ** *p* < 0.05.

Comparison of energy expenditure, dependent on nutritional status of the men, revealed significantly higher energy expenditure in individuals with normal body weight compared to those who were overweight/obese. This was observed at fasting state and throughout the test after high-carbohydrate, normo-carbohydrate, and high-fat meal challenge tests (Figure 2 and Figure 3). Also, the areas under the energy expenditure curve were higher in people with normal body weight during all meals test.

Next, we analyzed glucose oxidation after an HC meal and NC meal in Group I. In males with normal body weight (N1), glucose oxidation was significantly higher after the HC meal compared to NC meal at 120 (1.678 ± 0.189 vs. 1.059 ± 0.135 mg/kg/min, *p* = 0.01) and 180 min of the test (1.670 ± 0.200 vs. 0.793 ± 0.117 mg/kg/min, *p* = 0.0007) (Table 4). In overweight/obese people (O/O1), there was a tendency for greater glucose oxidation from 120 min after the HC meal in comparison to the NC meal (this difference was significant at 180 min of the study, and there was a trend at 240 min.) (Table 4). In subjects with normal body weight, the area under the glucose oxidation curve was significantly higher after an HC meal compared to the value of the area after an NC meal intake (*p* = 0.04). However, in overweight/obese people, there were no significant differences in the values of the areas under the glucose oxidation curves after these meals (Table 4).

In Group II, males with a normal body weight (N2) showed a tendency for higher glucose oxidation after the HC meal vs. HF meal at 120 min (1.007 ± 0.192 vs. 0.473 ± 0.177 mg/kg/min, *p* = 0.07), which was observed also in overweight/obese people (O/O2) at 180 min (0.850 ± 0.198 vs. 0.404 ± 0.126 mg/kg/min, *p* = 0.09) (Table 4). There were no significant differences in the areas under the glucose oxidation curves after an HC and HF meal (Table 4).

**Figure 4:**
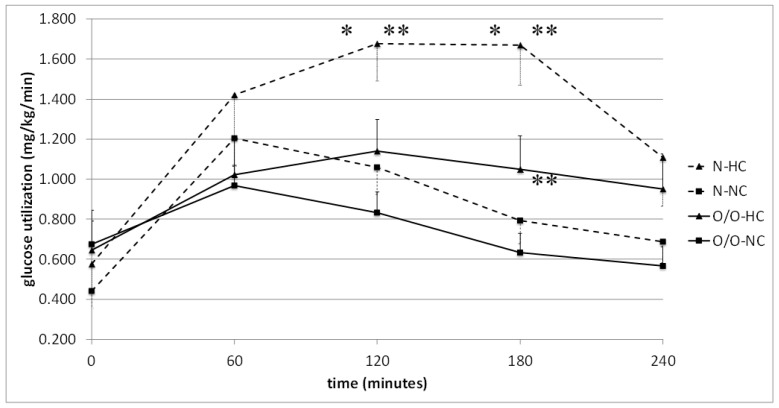
Glucose utilization (mg/kg/min) in people with normal body weight (N, the broken line) and overweight/obese people (O/O, the solid line) in fasting state (time 0 min) and after consumption (time 60–240 min) of a high-carbohydrate meal (HC) and a normo-carbohydrate meal (NC). The results are presented as mean values ± SE. The comparison between study groups N and O/O * *p* < 0.05; the comparison between meals HC and NC ** *p* < 0.05.

**Figure 5:**
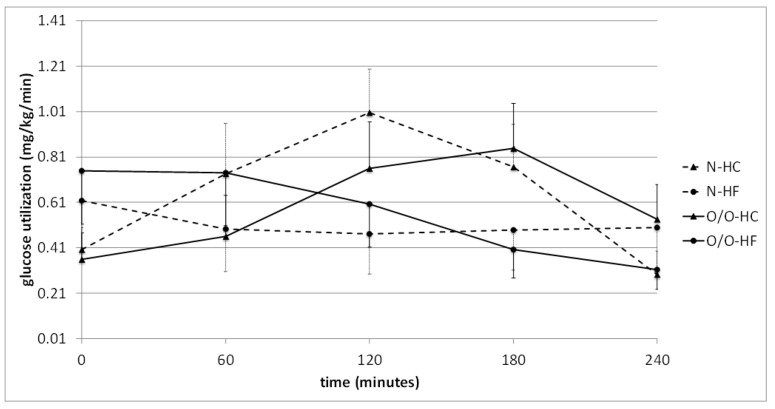
Glucose utilization (mg/kg/min) in people with normal body weight (N, the broken line) and overweight/obese people (O/O, the solid line) in fasting state (time 0 min) and after consumption (time 60–240 min) of a high-carbohydrate meal (HC) and a high-fat meal (HF). The results are presented as mean values ± SE. The comparison between study groups N and O/O; the comparison between meals HC and HF.

Then, a comparison of glucose oxidation in men with different BMIs was performed (Figure 4 and Figure 5) and it was found that glucose utilization at fasting state did not differ between men with normal body weight and overweight/obese people. In contrast, people with normal body weight (N1) showed significantly higher glucose oxidation after an HC meal at 120 and 180 min, and a tendency for higher glucose utilization at 60, 120, and 180 min after the NC meal compared to overweight/obese people (O/O1) (Figure 4). In men with a normal body weight (N1), after a high-carbohydrate meal intake, there was also a tendency towards increasing values of areas under the glucose oxidation curve compared to overweight/obese people (O/O1), which was not noted after a normo-carbohydrate meal. In Group II, no significant differences were found in the oxidation of glucose between subjects with normal body weight (N2) and overweight/obese people (O/O2) (Figure 5). A tendency towards greater glucose utilization was observed in people with N2 at 60 min after the HC meal compared to people with O/O2. Also observed was a tendency towards a higher value of the area under the glucose oxidation curve after a high-fat meal in overweight/obese subjects (O/O2) compared to people with normal body weight (N2).

Our next analysis showed that in Group I in men with normal body weight (N1) there was significantly higher lipid oxidation after the NC meal compared to HC meal at 120 and 180 min, and this trend was maintained at 240 min of the test (Table 5). Similar results were obtained in overweight/obese people (O/O1) (Table 5). After a normo-carbohydrate meal, both in normal body weight subjects and in overweight/obese people, area under the lipid oxidation curve was significantly higher than after a high-carbohydrate meal was observed (Table 5).

In contrast, in Group II, when comparing the HC meal with HF meal in people with normal body weight (N1), no significant differences in lipid oxidation were observed (Table 5). In overweight/obese subjects (O/O2), there was a tendency for greater lipid oxidation at fasting state and 60 min after the HC vs. HF meal intake, however, at 180 min of the study, lipid oxidation was significantly higher after a high-fat meal (Table 5). No significant differences, depending on the body weight, in the values of the areas under the curves of lipid oxidation after HC and HF meals were observed (Table 5).

**Figure 6:**
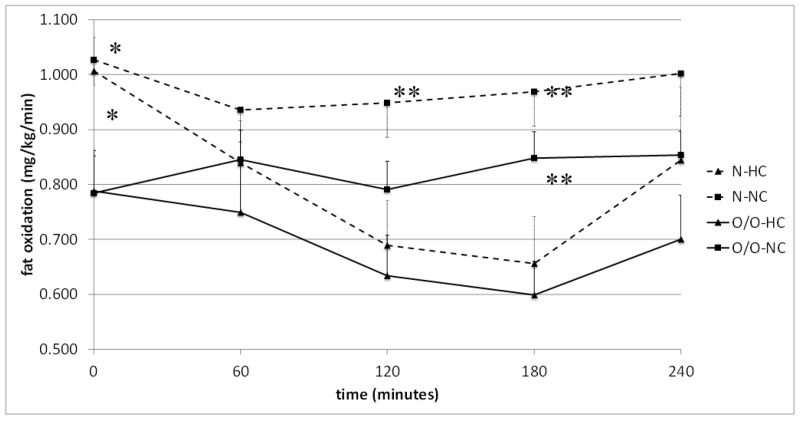
Fat oxidation (mg/kg/min) in people with normal body weight (N, the broken line) and overweight/obese people (O/O, the solid line) in fasting state (time 0 min) and after consumption (time 60–240 min) of a high-carbohydrate meal (HC) and a normo-carbohydrate meal (NC). The results are presented as mean values ± SE. The comparison between study groups N and O/O * *p* < 0.05; the comparison between meals HC and NC ** *p* < 0.05.

**Figure 7:**
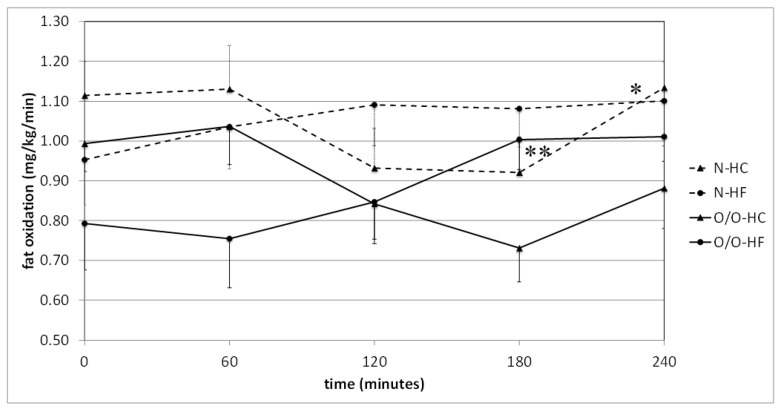
Fat oxidation (mg/kg/min) in people with normal body weight (N, the broken line) and overweight/obese people (O/O, the solid line) in fasting state (time 0 min) and after consumption (time 60–240 min) of a high-carbohydrate meal (HC) and a high-fat meal (HF). The results are presented as mean values ± SE. The comparison between study groups N and O/O * *p* < 0.05; the comparison between meals HC and HF ** *p* < 0.05.

Comparing the level of lipid oxidation, depending on the nutritional status of the examined men, it was found that at fasting state in Group I, it was significantly higher in people with normal body weight (N1) compared to overweight/obese individuals (O/O1) (1.007 ± 0.061 vs. 0.788 ± 0.074 mg/kg/min, *p* = 0.03 and 1.028 ± 0.047 vs. 0.784 ± 0.068 mg/kg/min, *p* = 0.009) (Figure 6). The tendency for higher lipid oxidation was maintained at 120, 180, and 240 min after the NC meal, which was not found after the HC meal. The area under the lipid oxidation curve was higher in N1 subjects than in O/O1 subjects only after a normo-carbohydrate meal. In Group II, no significant differences were observed in the oxidation of fasting lipids between men with different BMIs (Figure 7). However, after an HC meal at 180 min, a trend was noticed, and at 240 min, significantly higher lipid oxidation in people with normal body weight (N2) was observed. After an HF meal, the tendency towards higher lipid oxidation in subjects with normal body weight (N2) persisted throughout the study. There was also a trend towards higher values of the area under the lipid oxidation curve in subjects with normal body weight (N2) after HC meal and HF meal intake, compared to overweight/obese people (O/O2).

## 4. Discussion

The underlying cause of body fat accumulation is excessive energy supply in relation to its expenditure. There is no doubt that genetic factors determine the predisposition for the occurrence of obesity [26], but at the same time, a number of studies indicate that defining obesity as a genetically determined disease seems to have limited justification. The phenotype is a result of the genetic profile and the influence of external factors, whereas the occurrence of obesity outside the genetic factor is conditioned by the coexistence of the environmental factors, including an improper diet [27]. In the present study, we have found the differences in postprandial energy expenditure and substrates utilization between normal weight and overweight/obese men. Moreover, we have analyzed the results after intake of three meals with varying macronutrient content. Our experiments have shown that energy expenditure was significantly higher in people with normal body weight, compared to overweight/obese people, both at fasting state as well as throughout the study. In the literature, there are observations indicating higher energy expenditure in people with obesity [28]. The difference between the results most often is linked to the fact that they are presented in various units, in kcal/min, kcal/day, kcal/kg of lean body weight/min, or in kcal/kg of lean body weight/day, while in our work, the results are expressed in kcal/kg of body weight/min. The changes in energy expenditure observed after each of the meals were similar in people with normal body weight and in overweight/obese individuals. Other researchers have also shown that the postprandial increase in energy expenditure does not differ between normal body weight and overweight/obese people [9,10,11]. However, Marques-Lopes et al. [29] have observed that after a high-carbohydrate meal intake, a tendency towards higher energy expenditure exists in overweight people. The authors also reported that the respiratory quotient was higher than 1.0, indicating the activity of the lipogenesis pathway.

When we compared the energy expenditure levels between an HC and NC meal, no significant differences in the lean individuals were observed, except the trend towards a higher value of the area under the energy expenditure curve and higher energy expenditure at 180 min after a high-carbohydrate meal intake. In contrast, in the overweight/obese group, a tendency for higher energy expenditure was observed 60 min after a normo-carbohydrate meal. Comparing energy expenditure after HC and HF meal intake, we noted that it was higher after a high-carbohydrate meal intake in normal-weight and in overweight/obese group, but without any differences in the areas under the curves. Higher energy expenditure after a high-carbohydrate meal compared to a high-fat meal in people with normal body weight was also demonstrated by Raben et al. [30], however, after 4 h of testing the trend was reversed. Also, Tentolouris et al. [9] showed that due to increased activity of the sympathetic nervous system, energy expenditure is higher after a high-carbohydrate meal than after a high-fat meal, but it is comparable in people with normal body weight and overweight/obese people. Research by Potter et al. [31] showed that high-carbohydrate and mixed meals can affect the energy expenditure by activating the sympathetic nervous system, which was not observed after a high-fat meal.

The glucose utilization at fasting state did not differ between normal body weight and overweight/obese people. However, in the postprandial period, glucose utilization was significantly higher in subjects with normal body weight (N1) compared to overweight/obese people (O/O1) after a high-carbohydrate and normo-carbohydrate meal intake. What is surprising is that we did not observe these differences in Group II; only some trends were observed. Other researchers have shown that after high-carbohydrate and high-fat meal intake, the glucose oxidation does not differ in people with different body weight [9,10,11]. The higher glucose utilization observed in our study in people with normal body weight after HC meals may be related to the metabolic flexibility—the ability of the body to adapt and oxidize energy substrates depending on their availability. In overweight/obese people, there may be limited adaptive abilities of the body, characterized by impaired lipid oxidation at fasting state and disturbances in glucose utilization in the postprandial period when glucose availability increases, due to impaired insulin action [3].

The comparison between meals showed significantly higher glucose utilization after the HC meal than that after NC meal intake in normal body weight men. However, in overweight/obese people, there was only a tendency towards higher glucose uptake after high-carbohydrate meal intake, and again only some tendencies were observed in Group II. Also, other authors [30] have demonstrated the highest glucose utilization after a high-carbohydrate meal in a group of people with normal body weight. Our results are in line also with the study conducted by Tentolouris et al. [10], who showed that glucose utilization increased after a high-carbohydrate meal, whereas after a high-fat meal it was reduced in the group of people with normal body weight, as well as in obese individuals, as observed in this study.

Lipid oxidation analysis revealed higher fasting lipid oxidation in people with normal body weight (N1) compared to overweight/obese people (O/O1). After normo-carbohydrate meal intake, higher lipid oxidation was observed in people with normal body weight, probably due to higher fasting lipid oxidation, because the average lipid utilization values in people with normal body weight decreased, whereas in overweight/obese people, an increase in relation to the fasting value was observed. Also, in Group II, we observed the tendency for greater lipid oxidation in subjects with normal body weight (N2) compared to overweight/obese people (O/O2). Other researchers have shown that after a high-carbohydrate and high-fat meal, lipid oxidation does not differ between normal body weight and overweight/obese people [9,10,11]. Trends shown in our study, as in the case of glucose oxidation, may indicate an impaired metabolic flexibility [3], since in the fasting state—when the primary source of energy is free fatty acids derived from lipolysis of adipose tissue—overweight/obese subjects were characterized by lower lipid oxidation.

When we compared lipid oxidation between meals, we found that it was higher after the NC meal compared to the HC meal in both groups. In contrast, in men with normal body weight, comparison of lipid oxidation after the high-carbohydrate and high-fat meal revealed a significant reduction in lipid oxidation after a high-carbohydrate meal and increased oxidation after a high-fat meal, however, the differences were not statistically significant. Also, the authors [30] have shown that the consumption of a high-carbohydrate meal reduces the lipid oxidation in groups of people with normal body weight, whereas Tentolouris et al. [10] have demonstrated that the oxidation of lipids is reduced after a high-carbohydrate meal, both in people with normal body weight and in obese subjects. Reduced oxidation of fatty acids in the postprandial period is associated with an increase in body fat content, which has been confirmed in human studies [32]. One of the protective mechanisms against excessive accumulation of fat is the increase in fat oxidation during a high-fat diet, and it appears that in obese people these mechanisms are disturbed, which has been confirmed by other researchers [33]. However, Bergouignan et al. have shown that the ability to increase fat oxidation when using a high-fat diet is comparable for people with normal body weight and obesity [34]. In our study, we have noted that after a high-fat meal, lipid oxidation started to increase from 60 min and it was higher than after the high-carbohydrate meal intake. The observed tendency for greater lipid oxidation in overweight/obese men at fasting state and after a high-carbohydrate meal intake, when glucose is available as a potential energy substrate, may be another argument in favor of impaired metabolic flexibility in overweight/obese people [3].

The major strength of our study is that tests with two different meals were conducted in the same study group, and that we have used meals with the standardized macronutrient content. On the other hand, the major limitation of our study is that it was quite difficult to find volunteers who agreed to participate in all three meal tests, since the presented study is just a part of the larger project [14,15,16,35] and the protocol was very long and laborious. The other limitation is that normal-weight men in Group 1 were a little bit younger than overweight/obese individuals. Nevertheless, based on the conducted experiment, we can conclude that the fasting differences observed in overweight/obese men, together with the demonstrated limited ability of the body to adapt quickly to oxidize energy substrates, depending on their availability, indicate and confirm that these people are characterized by lower metabolic flexibility, which may be a result of increased adipose tissue and can lead to obesity-related diseases.

## 5. Conclusions

In the summary, we noted that energy expenditure was higher in people with normal body weight than in overweight/obese individuals at fasting state, as well as postprandially. The energy expenditure was higher after the high-carbohydrate meal in men with a normal body weight, whereas in overweight/obese people after a normo-carbohydrate meal intake. The lowest energy expenditure was noted after the high-fat meal intake in people with normal body weight and in overweight/obese men. The highest glucose utilization was demonstrated after a high-carbohydrate meal, and in people with normal body weight it was higher than in overweight/obese individuals. Surprisingly, in overweight/obese men, we observed higher glucose utilization up to 180 min after high-fat meal intake. At that time point, a significant increase in lipid oxidation was observed, whereas lipid oxidation started increasing at 60 min of test for people with normal body weight.

## Figures and Tables

**Table 1:** The study population characteristic. The results are presented as mean values ± SE. BMI—Body Mass Index.

Parameter		Normal-Weight	Overweight/Obese	*p*-Value
		Men	Men	
Group 1	*n*	11	12	
	Age (years)	33 ± 2	40 ± 2	0.01
	BMI	23.8 ± 0.5	31.4 ± 1.5	0.0002
	Body fat content (%)	17.9 ± 1.0	28.6 ± 1.7	0.00003
Group 2	*n*	12	11	
	Age (years)	33 ± 3	36 ± 3	0.24
	BMI	23.9 ± 0.2	33.7 ± 2.2	0.000001
	Body fat content (%)	18.6 ± 1.5	31.9 ± 2.7	0.0002

**Table 2:** The energy and macronutrients composition of meals.

Parameter	High-Carbohydrate Meal	Normo-Carbohydrate Meal	High-Fat Meal
Energy (kcal)	450	450	450
Carbohydrate (g)	100.5	51.1	4.0
Carbohydrate (% of total energy)	89.3	45.1	4.0
Fat (g)	0	12.6	47.5
Fat (% of total energy)	0	25.2	96
Protein (g)	12	36	0
Protein (% of total energy)	10.7	29.7	0
Fiber (g)	0	0.1	0

**Table 3:** Energy expenditure (kcal/kg/min) in men from Group I (G1, with normal body weight N1 and with overweight/obesity O/O1) on fasting (0 min) and in crossover trials after intake (60–240 min) of high carbohydrate (HC) meal and normo-carbohydrate (NC) meal and men from Group II (G2, with normal body weight N2 and with overweight/obesity O/O2) on fasting (0 min) and in crossover trials after intake (30–240 min) of high carbohydrate (HC) meal and high fat (HF) meal.

**G1**	**Time (min)**	**0**	**60**	**120**	**180**	**240**	**The Area under the Curve**
High-carbohydrate (HC) meal vs. normo- carbohydrate (NC) meal	N1-HC	0.0156 ± 0.0004	0.0176 ± 0.0006	0.0172 ± 0.0005	0.0168 ± 0.0005	0.0162 ± 0.0005	4.0689 ± 0.1191
	N1-NC	0.0152 ± 0.0004	0.0175 ± 0.004	0.0171 ± 0.0004	0.0162 ± 0.0004	0.0160 ± 0.0005	3.9562 ± 0.0973
	* *p* =	0.29	0.68	0.78	0.11	0.72	0.11
	O/O1-HC	0.00129 ± 0.0005	0.0143 ± 0.0004	0.0137 ± 0.0004	0.0129 ± 0.005	0.0134 ± 0.004	3.2434 ± 0.0918
	O/O1-NC	0.0131 ± 0.0004	0.0150 ± 0.0005	0.0139 ± 0.0004	0.0135 ± 0.0005	0.0134 ± 0.0005	3.3340 ± 0.0974
	* *p* =	0.70	0.70	0.72	0.27	0.83	0.21
**G2**	**Time (min)**	**0**	**60**	**120**	**180**	**240**	**The Area under the Curve**
High-carbohydrate (HC) meal vs. high-fat (HF) meal	N2-HC	0.0155 ± 0.0011	0.0172 ± 0.0008	0.0167 ± 0.0007	0.0156 ± 0.0008	0.0155 ± 0.0006	3.9040 ± 0.1864
	N2-HF	0.0151 ± 0.0005	0.0152 ± 0.0005	0.0157 ± 0.0005	0.0155 ± 0.0004	0.0160 ± 0.0006	3.7185 ± 0.1190
	** *p* =	0.68	0.01	0.11	0.81	0.19	0.20
	O/O2-HC	0.0133 ± 0.0004	0.0145 ± 0.0005	0.0139 ± 0.0003	0.0131 ± 0.0004	0.0132 ± 0.0004	3.2887 ± 0.0960
	O/O2-HF	0.0132 ± 0.0005	0.0128 ± 0.0004	0.0132 ± 0.0004	0.0137 ± 0.0005	0.0134 ± 0.0005	3.1815 ± 0.1023
	** *p* =	0.78	0.0008	0.18	0.24	0.46	0.23

* The results are presented as mean values ± SE for the comparison between HC and NC. ** The results are presented as mean values ± SE for the comparison between HC and HF.

**Table 4:** Glucose utilization (mg/kg/min) in men from Group I (G1, with normal body weight N1 and with overweight/obesity O/O1) on fasting (0 min) and in crossover trials after intake (60–240 min) of high-carbohydrate (HC) meal and normo-carbohydrate (NC) meal and men from Group II (G2, with normal body weight N2 and with overweight/obesity O/O2) on fasting (0 min) and in crossover trials after intake (30–240 min) of high-carbohydrate (HC) meal and high-fat (HF) meal.

**G1**	**Time (min)**	**0**	**60**	**120**	**180**	**240**	**The Area under the Curve**
High-carbohydrate (HC) meal vs. normo- carbohydrate (NC) meal	N1-HC	0.575 ± 0.126	1.420 ± 0.222	1.678 ± 0.189	1.670 ± 0.200	1.109 ± 0.245	344 ± 38
	N1-NC	0.441 ± 0.085	1.205 ± 0.140	1.059 ± 0.135	0.793 ± 0.117	0.687 ± 0.103	249 ± 23
	* *p* =	0.15	0.32	0.01	0.0007	0.19	0.04
	O/O1-HC	0.645 ± 0.144	1.023 ± 0.174	1.140 ± 0.159	1.049 ± 0.169	0.950 ± 0.176	241 ± 33
	O/O1-NC	0.676 ± 0.169	0.968 ± 0.102	0.833 ± 0.103	0.632 ± 0.096	0.566 ± 0.096	255 ± 19
	* *p* =	0.85	0.80	0.08	0.05	0.06	0.67
**G2**	**Time (min)**	**0**	**60**	**120**	**180**	**240**	**The Area under the Curve**
High-carbohydrate (HC) meal vs. high-fat (HF) meal	N2-HC	0.404 ± 0.097	0.738 ± 0.222	1.007 ± 0.192	0.768 ± 0.188	0.295 ± 0.103	178 ± 38
	N2-HF	0.619 ± 0.215	0.495 ± 0.187	0.473 ± 0.177	0.491 ± 0.177	0.501 ± 0.200	151 ± 55
	** *p* =	0.31	0.50	0.07	0.33	0.57	0.84
	O/O2-HC	0.361 ± 0.117	0.461 ± 0.181	0.761 ± 0.205	0.850 ± 0.198	0.537 ± 0.154	151 ± 41
	O/O2-HF	0.752 ± 0.235	0.743 ± 0.234	0.605 ± 0.191	0.404 ± 0.126	0.315 ± 0.087	194 ± 39
	** *p* =	0.12	0.40	0.50	0.09	0.27	0.57

* The results are presented as mean values ± SE for the comparison between HC and NC. ** The results are presented as mean values ± SE for the comparison between HC and HF.

**Table 5:** Fat oxidation (mg/kg/min) in men from Group I (G1, with normal body weight N1 and with overweight/obesity O/O1) on fasting (0 min) and in crossover trials after intake (60–240 min) of high-carbohydrate (HC) meal and normo-carbohydrate (NC) meal and men from Group II (G2, with normal body weight N2 and with overweight/obesity O/O2) on fasting (0 min) and in crossover trials after intake (30–240 min) of high-carbohydrate (HC) meal and high-fat (HF) meal.

**G1**	**Time (min)**	**0**	**60**	**120**	**180**	**240**	**The Area under the Curve**
High-carbohydrate (HC) meal vs. normo- carbohydrate (NC) meal	N1-HC	1.007 ± 0.061	0.839 ± 0.077	0.689 ± 0.081	0.656 ± 0.086	0.844 ± 0.133	185 ± 18
	N1-NC	1.028 ± 0.047	0.936 ± 0.059	0.949 ± 0.062	0.969 ± 0.062	1.002 ± 0.078	234 ± 13
	* *p* =	0.60	0.28	0.009	0.003	0.12	0.03
	O/O1-HC	0.788 ± 0.074	0.749 ± 0.089	0.634 ± 0.074	0.598 ± 0.061	0.700 ± 0.080	167 ± 15
	O/O1-NC	0.784 ± 0.068	0.845 ± 0.054	0.791 ± 0.052	0.848 ± 0.048	0.854 ± 0.043	198 ± 8
	* *p* =	0.95	0.37	0.08	0.003	0.11	0.04
**G2**	**Time (min)**	**0**	**60**	**120**	**180**	**240**	**The Area under the Curve**
High-carbohydrate (HC) meal vs. high-fat (HF) meal	N2-HC	1.115 ± 0.086	1.330 ± 0.110	0.932 ± 0.099	0.921 ± 0.090	1.134 ± 0.065	247+22
	N2-HF	0.953 ± 0.113	1.035 ± 0.106	1.090 ± 0.104	1.081 ± 0.096	1.100 ± 0.113	254 ± 25
	** *p* =	0.32	0.46	0.27	0.20	0.77	0.96
	O/O2-HC	0.993 ± 0.070	1.036 ± 0.096	0.842 ± 0.089	0.731 ± 0.084	0.881 ± 0.101	214 ± 21
	O/O2-HF	0.792 ± 0.116	0.754 ± 0.123	0.847 ± 0.105	1.003 ± 0.075	1.010 ± 0.062	210 ± 18
	** *p* =	0.16	0.13	0.83	0.02	0.02	0.95

* The results are presented as mean values ± SE for the comparison between HC and NC. ** The results are presented as mean values ± SE for the comparison between HC and HF.
